# Cardiac Arrest Care on United States Golf Courses—Up to Par Yet?

**DOI:** 10.1016/j.acepjo.2025.100278

**Published:** 2025-11-25

**Authors:** Bret Gustafson, Brett Todd, Peter Prescott, Yuying Xing, Bryan McNally, Man Qi, Rabab Al-Araji, Robert Swor, CARES Surveillance Group

**Affiliations:** 1 Department of Emergency Medicine, Corewell Health East William Beaumont University Hospital, Royal Oak, Michigan, USA; 2 Oakland University William Beaumont School of Medicine, Rochester, Michigan, USA; 3 Division of Biostatistics, Corewell Health Research Institute, Grand Rapids, Michigan, USA; 4 Emory University School of Medicine, Atlanta, Georgia, USA; 5 Rollins School of Public Health, Atlanta, Georgia, USA

**Keywords:** cardiac arrest, automated external defibrillation, recreational facilities, bystander CPR

## Abstract

Out-of-hospital cardiac arrest (OHCA) is a significant public health issue with a <10% survival rate. Although exercise is beneficial for cardiovascular health, it is also associated with increased risk of OHCA during and immediately after exertion. Golf courses present the fifth most frequent location for OHCA in public settings. Bystander interventions, such as cardiopulmonary resuscitation (CPR) and automated external defibrillator (AED) use, are crucial for improving survival rates. Despite recommendations from organizations like the Professional Golfers’ Association of America recommending AEDs at golfing facilities, AED availability and utilization on golf courses prior to Emergency Medical Services arrival remain unknown.

**Objectives:**

This investigation aimed to determine the incidence of OHCA on golf courses and to examine patient demographics, event and golf course characteristics, and their association with bystander CPR provision, AED use, and survival outcomes.

**Methods:**

This retrospective study utilized data from the Cardiac Arrest Registry to Enhance Survival from 2020 to 2023. Addresses of cardiac arrests in recreational facilities were spatially linked to a comprehensive list of golf course addresses using ArcGIS. Cases identified as occurring in a recreational location, within 400 m of both the incident address and a nearby golf course, were included in the analysis. The primary outcome was the frequency of bystander interventions for either CPR or AED use. Secondary outcomes included survival to hospital admission and discharge, and a favorable neurologic outcome (cerebral performance category 1 or 2). Descriptive statistics and univariate analyses were employed to examine patient demographics, golf course characteristics, bystander interventions, and patient outcomes. Multivariable regression analysis was also used to assess these associations.

**Results:**

A total of 476 OHCA cases were identified on golf courses during the study period. The patients were predominantly male (91.4%, *n* = 435) and White (85.1%, *n* = 330), with a mean age of 64.6 (SD 15.5). Most patients (61.8%) had an initial shockable rhythm.

Distribution of cases was not different by course geographic region, course size, or public vs private course. Overall survival to discharge was 30.5% (*n* = 145), with almost all survivors (97.2%, *n* = 141) having a good neurologic outcome. For patients with Utstein characteristics (witnessed, shockable rhythm) and who had an AED placed, survival to discharge was 63.8%.

AED use was associated with significantly increased survival to hospital discharge (37.9% vs 18.7% OR_adj_ 1.9 (95% CI, 1.1, 3.3, *P* <.05), with AEDs being used more frequently on private courses (32.1% vs 19.7%, *P* = .003).

**Conclusion:**

Patients experiencing OHCA on golf courses frequently receive bystander interventions, including both CPR and AED use. OHCA patients on golf courses had high survival rates and excellent neurologic outcomes. This study underscores the benefit of, and critical need for, initiatives promoting CPR and AED resources, as well as comprehensive CPR training and rapid response protocols at golf courses

**The Bottom Line**

Golf is a very popular sport, and golfers may be at risk for out-of-hospital cardiac arrest (OHCA). Little is known regarding OHCA events on golf courses, care rendered, and survival outcomes. We identified 476 OHCA cases that occurred on golf courses in the United States. Most patients received bystander cardiopulmonary resuscitation, and almost 25% had an automated external defibrillator placed. Survival was 30.5% (*n* = 145), with some subsets having survival rates as high as 63.8%. Virtually all survivors (97.2%, *n* = 141) had a favorable neurologic outcome. This study highlights the survival potential and gaps of optimal care for golf course OHCA patients.

## 1 Introduction

### 1.1 Background

Out-of-hospital cardiac arrest (OHCA) is an important public health issue, with more than estimated 350,000 OHCAs annually in the United States, and a survival to hospital discharge rate of <10%.^1^ This high incidence and low survival rate underline the need for rapid emergency interventions. Cardiac arrest (CA) is unique in that bystander actions, such as CPR and automated external defibrillator (AED) application by bystanders, double or triple survival.^2^^,^^3^

Physical exercise is universally recognized for its benefits on cardiovascular health, but it can act as a trigger for cardiac events, as OHCA risk increases during vigorous exercise, especially in older or unfit individuals.^4^, ^5^, ^6^ Recreational facilities are common locations for OHCA, where immediate CPR and bystander-initiated AED use significantly improve survival rates.^7^ For these reasons, many states have mandated CPR training for workers at recreational facilities and placement of AEDs in health clubs and recreational facilities. Typically, however, these statutes do not include outdoor recreational facilities.^8^

Golf is often perceived as a low-intensity outdoor activity and provides an opportunity for adults to engage in regular physical activity, potentially mitigating some cardiovascular risk factors.^9^ Nevertheless, golf courses rank as the fifth most frequent location for OHCA in public settings.^10^ This paradoxical scenario underscores the necessity of addressing emergency preparedness and response specifically within these environments.

### 1.2 Importance

OHCAs at golf courses present unique survival challenges due to the remote location of many courses, distances from players to clubhouse facilities, and potential delays in activating 911 and emergency medical response. Lay and professional (golf and medical) publications have identified the need for AED placement at golfing facilities to address this problem. Advocacy efforts date back as far as 2005, with notable recommendations from various organizations, including a prominent magazine article in the United Kingdom and more recent endorsements from the Professional Golfers’ Association (PGA) of America, which suggest a minimum of 2 AEDs at golfing facilities.^10^^,^^11^ A recent infographic and CPR protocol have been developed for display at golf courses.^12^ However, the impact of these recommendations on actual AED use and survival outcomes remains unclear.

### 1.3 Goals of This Investigation

There is a paucity of research on this topic. Small studies from Pennsylvania and Michigan identify suboptimal preparedness for OHCA events, infrequent availability of AEDs at golf courses, and suboptimal use of available AEDs. This may be because of inadequate training of golf course staff or failure of lay bystanders to recognize CA.^13^^,^^14^ Data that characterizes OHCA care on golf courses is needed to identify the current status of such care, including bystander care, and outcomes of patients who suffer OHCA.

## 2 Objectives

This investigation aimed to determine the incidence of OHCA on golf courses and to examine patient demographics, event and golf course characteristics, and their association with bystander CPR provision, AED use, and survival outcomes.

## 3 Methods

### 3.1 Study Type

We performed a retrospective cohort study of OHCAs that occurred on golf courses from 2020 to 2023, using the Cardiac Arrest Registry to Enhance Survival (CARES) database to identify the study cohort.

### 3.2 Setting and Subjects

CARES is a large national OHCA database. In 2024, CARES reported cardiac arrest data from 37 state-based registries as well as data from 43 community sites in 11 additional states, representing a catchment area of approximately 186 million people or 56% of the US population.^7^ CARES has a robust data collection and auditing process to ensure the integrity and cleanliness of the data, which is described in detail in its annual report.^7^

### 3.3 Subjects

Candidate cases were identified from the parent CARES database. All cases identified as occurring in a recreational setting (CARES location type) were included. The incident address was linked with a commercial database of golf course locations.^15^ Cases were linked using radii of distance between incident and golf course addresses ranging from 200 to 1600 m using ArcGIS, with the optimal linkage radius determined by consensus of study authors. Because of the large footprint of golf courses and potential for variation in the identification of course addresses, the authors chose a 400 m radius for matching.

### 3.4 Outcomes

We report demographic variables, Emergency Medical Services (EMS) response variables, and whether bystander CPR was provided. For study purposes, a bystander is defined as a responder not part of the EMS system who provided CPR before EMS arrival. This would include lay bystanders as well as golf course personnel. We assessed whether an AED was applied, by whom, and whether an AED shock was provided. Survival outcomes include survival to hospital admission, survival to hospital discharge, and survival to discharge with good neurologic status (cerebral performance category of 1 or 2).

### 3.5 Statistical Analysis

Descriptive statistics were used to summarize both patient characteristics and other relevant factors. Continuous variables were presented as means with SDs or medians with interquartile ranges, whereas categoric variables were expressed as frequencies and percentages. The Fisher’s exact test was applied to analyze categoric variables, and the Mann-Whitney test was used for continuous variables.

Univariate logistic regression was performed to assess the associations between survival to hospital discharge and key clinical process variables. For the multivariable model, covariates were selected purposefully based on prior literature and established predictors of OHCA survival, including witnessed status, first rhythm, bystander CPR, AED use, patient demographics (age, sex, race/ethnicity), and course type (public vs private). We reported patient sex as was recorded by the treating EMS professional. To mitigate the risk of overfitting, given our sample size, variables with a *P* value <0.05 (bystander CPR, bystander AED, witness status, patient first rhythm type, and course type), along with patient demographic variables (age, gender, race, and ethnicity), were included in the multivariable logistic regression model. Models were assessed using variance inflation factors, Hosmer–Lemeshow goodness-of-fit, and receiver operating characteristic (ROC) curve analysis.

Odds ratios (OR) and adjusted odds ratios (aOR) were reported with 95% CIs and corresponding *P* values for all logistic regression analyses. All tests of statistical significance were 2-sided with a threshold of *P* < 0.05. Data analyses were conducted using R version 4.3.1 (R Foundation for Statistical Computing).

The study was approved under a waiver of authorization by the Beaumont Research Institute, IRB #2024-126.

## 4 Results

A total of 476 OHCA events were identified at 406 golf courses in the regions of the United States covered by the CARES database between 2020 and 2023. Patients were predominantly male (91.4%, *n* = 435) and White (85.1%, *n* = 330), with a mean age of 64.6 years (SD 15.5). An initial shockable rhythm was present in 61.8% of cases, and 68.3% of arrests were witnessed. Bystander cardiopulmonary resuscitation (CPR) was performed in 73.7% of cases (*n* = 351), and an AED was applied by a bystander in 24.6% (*n* = 117) (Table 1, Table S1).

**Table 1:** Patient demographics stratified by provision of public AED use.

Variables	No public AED use	Public AED use	Total cases	*P* value
	No (*N* = 359)	Yes (*N* = 117)	Total (*N* = 476)	
Age (y)				.311
Mean (SD)	63.9 (16.2)	66.8 (12.8)	64.6 (15.5)	
Median (Q1, Q3)	67.0 (58.0, 75.0)	67.0 (61.0, 75.0)	67.0 (59.0, 75.0)	
Age category				.239
≤18 y	8 (2.2%)	1 (0.9%)	9 (1.9%)	
19-34 y	17 (4.7%)	2 (1.7%)	19 (4.0%)	
35-49 y	27 (7.5%)	4 (3.4%)	31 (6.5%)	
50-64 y	103 (28.7%)	36 (30.8%)	139 (29.2%)	
65+ y	204 (56.8%)	74 (63.2%)	278 (58.4%)	
Sex				.261
Female	28 (7.8%)	13 (11.1%)	41 (8.6%)	
Male	331 (92.2%)	104 (88.9%)	435 (91.4%)	
RaceEthnicity				.365
Black	16 (5.5%)	5 (5.1%)	21 (5.4%)	
Hispanic/Latino	14 (4.8%)	3 (3.0%)	17 (4.4%)	
Other	18 (6.2%)	2 (2.0%)	20 (5.2%)	
White	241 (83.4%)	89 (89.9%)	330 (85.1%)	
Missing	70	18	88	
First rhythm type				.012
Nonshockable	149 (41.5%)	33 (28.2%)	182 (38.2%)	
Shockable	210 (58.5%)	84 (71.8%)	294 (61.8%)	
Bystander CPR?				<.001
No	122 (34.0%)	3 (2.6%)	125 (26.3%)	
Yes	237 (66.0%)	114 (97.4%)	351 (73.7%)	
Witness				.013
Unwitnessed	85 (23.7%)	15 (12.8%)	100 (21.0%)	
Witnessed	274 (76.3%)	102 (87.2%)	376 (79.0%)	
EMS response interval				.907
Mean (SD)	12.1 (6.6)	17.7 (60.4)	13.5 (30.8)	
Median (Q1, Q3)	10.7 (8.0, 14.6)	10.8 (8.7, 13.5)	10.8 (8.1, 14.1)	
Missing	70	20	90	
Survived to hospital discharge?				<.001
No	269 (74.9%)	62 (53.0%)	331 (69.5%)	
Yes	90 (25.1%)	55 (47.0%)	145 (30.5%)	
Golf course characteristics				
US region				.085
Midwest	74 (20.6%)	17 (14.5%)	91 (19.1%)	
Northeast	32 (8.9%)	10 (8.5%)	42 (8.8%)	
South	100 (27.9%)	47 (40.2%)	147 (30.9%)	
West	153 (42.6%)	43 (36.8%)	196 (41.2%)	
Holes				.099
>18	57 (16.0%)	21 (18.1%)	78 (16.5%)	
18	266 (74.5%)	91 (78.4%)	357 (75.5%)	
9	34 (9.5%)	4 (3.4%)	38 (8.0%)	
Missing	2	1	3	
Public/private golf course				.003
Private	127 (35.4%)	60 (51.3%)	187 (39.3%)	
Public	232 (64.6%)	57 (48.7%)	289 (60.7%)	

AED application occurred significantly more often on private courses than public courses (32.1% vs 19.7%, *P* = .003) and was more likely when the arrest was witnessed and bystander CPR was provided.

Of all patients, 53.4% (*n* = 254) survived to hospital admission. Overall survival to hospital discharge was 30.5% (*n* = 145). Among survivors, 97.2% (*n* = 141) were discharged with a favorable neurologic status, defined as a cerebral performance category score of 1 or 2. In the subset of patients who met Utstein criteria—witnessed arrest, initial shockable rhythm, as well as AED application—survival to hospital discharge was 63.8% (Table S2).

Significant predictors of survival in univariate analysis included bystander CPR, witnessed status, and initial shockable rhythm. In the multivariable analysis, as expected, AED use, witnessed arrest, and shockable initial rhythm were all associated with improved survival (Table 2). Arrests that occurred in public golf courses were less likely to survive to discharge compared to private golf courses.

**Table 2:** Multivariable outcomes associated with survival to hospital discharge.

Characteristic	Univariate				Multivariable		
	*N*	OR	95% CI	*P* value	aOR	95% CI	*P* value
Bystander CPR?	476						
No		ref					
Yes		2.21	1.37, 3.70	.002	1.06	0.55, 2.05	.900
Public AED use?	476						
No		ref					
Yes		2.65	1.72, 4.10	<.001	1.91	1.10, 3.33	.022
Witness	476						
Unwitnessed		ref					
Witnessed		4.01	2.20, 7.97	<.001	2.65	1.20, 6.55	.023
Age category 2	476						
≤18 y		ref					
19-34 y		0.92	0.17, 5.55	>.900	1.05	0.11, 12.5	>.900
35-49 y		1.26	0.28, 6.88	.800	1.14	0.14, 12.0	>.900
50-64 y		0.96	0.24, 4.70	>.900	0.52	0.08, 4.78	.500
65+ y		0.79	0.20, 3.83	.700	0.43	0.07, 3.96	.400
Gender	476						
Female		ref					
Male		0.94	0.48, 1.93	.900	1.33	0.52, 3.70	.600
Race ethnicity	388						
Black		ref					
Hispanic/Latino		0.83	0.20, 3.31	.800	1.04	0.20, 5.35	>.9
Other		0.50	0.11, 2.02	.300	0.46	0.08, 2.21	.300
White		0.89	0.36, 2.42	.800	0.74	0.26, 2.25	.600
First rhythm type	476						
Non-shockable		ref					
Shockable		6.93	4.14, 12.2	<.001	5.37	2.98, 10.2	<.001
Public/private golf course	476						
Private		ref					
Public		0.56	0.38, 0.84	.004	0.52	0.31, 0.87	.013

OR, odds ratio.

The variance inflation factor values for the multivariable logistic regression were all <2, indicating no multicollinearity among the variables. To evaluate the accuracy of predicting survival to hospital discharge, ROC curve analysis was performed. The area under the curve was 0.759, indicating a good ability of the model to distinguish between patients who survived to hospital discharge and those who did not. The Hosmer-Lemeshow goodness-of-fit test was used to assess the fit of the multivariable regression model, with a *P* value of .336 indicating a good model fit.

## 5 Limitations

Several limitations must be acknowledged. Our method of geospatial linkage based on proximity to golf course addresses may have misclassified some cases, especially for large properties or shared addresses, although we used recreational location type as a case qualifier. We could not independently verify whether the CA occurred during golf play or another event on the course grounds. Although our data identify cases in which an AED was used, we are unable to determine if an AED was available but not used. Operational questions regarding device and personnel availability, personnel training, communications with course and EMS personnel, and other factors necessary to provide emergency care when needed are beyond the scope of this study, but need to be addressed to improve survival.

Regarding the statistical analysis, our study was primarily descriptive, and the multivariable regression should be interpreted as exploratory. We limited covariate inclusion to well-established predictors of OHCA survival (witnessed status, initial rhythm, bystander CPR, AED use, demographics, and course type) to avoid overfitting, given the modest number of events. Important factors such as EMS response interval were not consistently available in CARES and therefore could not be incorporated, which may have resulted in residual confounding. Although model diagnostics suggested good calibration and discrimination, the regression results should be viewed as hypothesis-generating and supportive rather than definitive. Our univariate analysis identified a survival benefit of bystander CPR, but not in the multivariable analysis. We attribute this finding to the small sample size and comparatively less robust survival impact of CPR compared with AED utilization. Finally, although CARES is a comprehensive registry, it does not capture all US OHCAs, and our findings may not be generalizable to nonparticipating regions.

## 6 Discussion

This study represents the first large analysis of OHCA occurring on golf courses in the United States. Our findings demonstrate that patients experiencing CA in these settings frequently receive bystander interventions, including CPR and use of AEDs. These early actions are associated with improved survival to hospital discharge and favorable neurologic outcomes.

The overall survival to hospital discharge in our cohort was 30.5%, substantially higher than the national average of 10.5% reported for OHCAs across all settings in 2024.^7^ Among patients with Utstein characteristics—witnessed arrest, shockable initial rhythm, as well as receipt of AED placement—survival approached 64%, further emphasizing the critical role of early recognition and intervention. Given that golf is typically a group activity, we would expect this population to have a high rate of quickly recognized events, enhancing survival. These results are consistent with prior literature demonstrating that survival from OHCA is highly dependent on rapid CPR and defibrillation, ideally within minutes of collapse.

Despite strong evidence and longstanding recommendations from national organizations such as the PGA of America, bystander AED placement occurred in 25% of cases. Although this percentage compares favorably to bystander AED reported in large databases, it is similar to reports of bystander AED use in other recreational facilities. Kolkailah et al^16^ reported in a national study of bystander use of AEDs in recreational facilities by rate of 19.0% (median) (7.4%-50.0%, range). This work also looked at variation in AED use by whether or not a state had legislation mandating recreational facility AED placement. They identified no difference in statewide public AED use stratified by the presence of a law mandating the placement of AEDs in recreational facilities.^16^ Compared with golf courses and recreational sites, CARES data reports AED use varies in public location by state (5.8%-18.2%).^17^ These data do not stratify AED use by other public facilities, which commonly have public AEDs (airports, casinos, governmental buildings, etc). Our summary of this literature suggests that legislation (recreational facilities), and professional organization advocacy (such as golf courses) without local actions, moderately improve AED provision at the location of a CA.

These findings highlight an important implementation gap: although the survival benefit of AED use is unequivocal, many golf courses may still lack the equipment, training, or protocols needed to ensure timely deployment. Barriers such as large course footprints, long distances between play areas and clubhouses, or a lack of signage may further impede rapid response. These challenges underscore the need for targeted interventions—such as strategic AED placement throughout courses, mobile AED units with course staff (eg, maintenance, refreshment carts, course marshals), and standardized emergency response protocols tailored to golf course layouts. Single-course examples of integrated programs, including the use of in situ simulations, have been published online.^17^^,^^18^ Our observation that AEDs were more likely to be used at private courses than public ones (32.1% vs 19.7%) suggests that the availability of resources, such as differences in staffing, training, and equipment at private courses, results in better outcomes.

Our results also have policy implications. Although many states have mandated AEDs in indoor recreational facilities, these requirements often do not address outdoor venues like golf courses. Given that golf courses rank as a relatively more common public location for OHCA, updating legislation to include these settings may have a meaningful public health impact. Additionally, educational initiatives aimed at training staff and patrons in CPR and AED use could further close the gap between potential and realized benefit.

Compared with other recreational activities, patients experiencing OHCA on United States golf courses frequently receive bystander interventions, including both CPR and AED use. This immediate action is associated with high survival rates and excellent neurologic outcomes. This study underscores the benefit of, and critical need for, initiatives promoting CPR and AED resources as well as comprehensive CPR training and rapid response protocols at golf courses.

## Author Contributions

RS, BG, and BT conceived and designed the study. BG and PP performed the literature search. RA and MQ performed the data extraction and data linkage. RS and YX managed the data. YX analyzed the data. RS, RG, BT, and PP drafted the manuscript, and all authors contributed substantially to its revision. BM provided content expertise about the dataset and topic. RS takes responsibility for the paper as a whole.

## Funding and Support

By *JACEP Open* policy, all authors are required to disclose any and all commercial, financial, and other relationships in any way related to the subject of this article as per ICMJE conflict of interest guidelines (see www.icmje.org). The authors have stated that no such relationships exist.

## Conflict of Interest

All authors have affirmed they have no conflicts of interest to declare.
