# Supplementary material for: Cardiac Arrest Care on United States Golf Courses—Up to Par Yet?
Source: J Am Coll Emerg Physicians Open. 2025 Nov 25;7(1):100278. doi: 10.1016/j.acepjo.2025.100278 (PMC12686694; doi:10.1016/j.acepjo.2025.100278)
Supplement: Supplementary Tables 1 and 2 [file mmc1.docx]

Supplemental Table 1

|  | **No Bystander CPR** | **Bystander CPR** | **Total Cases** |  |
| --- | --- | --- | --- | --- |
|  | No (N=125) | Yes (N=351) | Total (N=476) | p value |
| **Age (Years)** |  |  |  | 0.051 |
| **Mean (SD)** | 61.9 (17.6) | 65.6 (14.6) | 64.6 (15.5) |  |
| **Median (Q1, Q3)** | 65.0 (51.0, 74.0) | 67.0 (60.0, 75.0) | 67.0 (59.0, 75.0) |  |
| **Age Category** |  |  |  | 0.017 |
| **<=18** | 3 (2.4%) | 6 (1.7%) | 9 (1.9%) |  |
| **19-34** | 8 (6.4%) | 11 (3.1%) | 19 (4.0%) |  |
| **35-49** | 15 (12.0%) | 16 (4.6%) | 31 (6.5%) |  |
| **50-64** | 35 (28.0%) | 104 (29.6%) | 139 (29.2%) |  |
| **65+** | 64 (51.2%) | 214 (61.0%) | 278 (58.4%) |  |
| **Gender** |  |  |  | 0.195 |
| **Female** | 7 (5.6%) | 34 (9.7%) | 41 (8.6%) |  |
| **Male** | 118 (94.4%) | 317 (90.3%) | 435 (91.4%) |  |
| **RaceEthnicity** |  |  |  | 0.175 |
| **Black** | 4 (4.1%) | 17 (5.9%) | 21 (5.4%) |  |
| **Hispanic/Latino** | 8 (8.2%) | 9 (3.1%) | 17 (4.4%) |  |
| **Other** | 6 (6.1%) | 14 (4.8%) | 20 (5.2%) |  |
| **White** | 80 (81.6%) | 250 (86.2%) | 330 (85.1%) |  |
| **Missing** | 27 | 61 | 88 |  |
| **US Region** |  |  |  | 0.684 |
| **Midwest** | 27 (21.6%) | 64 (18.2%) | 91 (19.1%) |  |
| **Northeast** | 12 (9.6%) | 30 (8.5%) | 42 (8.8%) |  |
| **South** | 34 (27.2%) | 113 (32.2%) | 147 (30.9%) |  |
| **West** | 52 (41.6%) | 144 (41.0%) | 196 (41.2%) |  |
| **Public AED Use?** |  |  |  | < 0.001 |
| **No** | 122 (97.6%) | 237 (67.5%) | 359 (75.4%) |  |
| **Yes** | 3 (2.4%) | 114 (32.5%) | 117 (24.6%) |  |
| **First Rhythm Type** |  |  |  | < 0.001 |
| **Non-shockable** | 68 (54.4%) | 114 (32.5%) | 182 (38.2%) |  |
| **Shockable** | 57 (45.6%) | 237 (67.5%) | 294 (61.8%) |  |
| **Holes** |  |  |  | 0.074 |
| **>18** | 18 (14.5%) | 60 (17.2%) | 78 (16.5%) |  |
| **18** | 90 (72.6%) | 267 (76.5%) | 357 (75.5%) |  |
| **9** | 16 (12.9%) | 22 (6.3%) | 38 (8.0%) |  |
| **Missing** | 1 | 2 | 3 |  |
| **PublicPrivate Golf Course** |  |  |  | 0.671 |
| **Private** | 47 (37.6%) | 140 (39.9%) | 187 (39.3%) |  |
| **Public** | 78 (62.4%) | 211 (60.1%) | 289 (60.7%) |  |
| **Witness** |  |  |  | < 0.001 |
| **Unwitnessed** | 51 (40.8%) | 49 (14.0%) | 100 (21.0%) |  |
| **Witnessed** | 74 (59.2%) | 302 (86.0%) | 376 (79.0%) |  |
| **EMS Response Interval** |  |  |  | 0.919 |
| **Mean (SD)** | 11.8 (6.3) | 14.1 (35.8) | 13.5 (30.8) |  |
| **Median (Q1, Q3)** | 11.1 (8.1, 14.9) | 10.6 (8.3, 13.8) | 10.8 (8.1, 14.1) |  |
| **Missing** | 22 | 68 | 90 |  |

Supplemental Table 2 Survival to Hospital Discharge and Patient Demographics

|  | **Died** | **Survival to Discharge** |  |  |
| --- | --- | --- | --- | --- |
|  | No (N=331) | Yes (N=145) | Total (N=476) | p value |
| **Age (Years)** |  |  |  | 0.105 |
| **Mean (SD)** | 65.4 (15.5) | 62.9 (154) | 64.6 (15.5) |  |
| **Median (Q1, Q3)** | 68.0 (59.0, 75.0) | 66.0 (59.0, 73.0) | 67.0 (59.0, 75.0) |  |
| **Age Category** |  |  |  | 0.729 |
| **<=18** | 6 (1.8%) | 3 (2.1%) | 9 (1.9%) |  |
| **19-34** | 13 (3.9%) | 6 (4.1%) | 19 (4.0%) |  |
| **35-49** | 19 (5.7%) | 12 (8.3%) | 31 (6.5%) |  |
| **50-64** | 94 (28.4%) | 45 (31.0%) | 139 (29.2%) |  |
| **65+** | 199 (60.1%) | 79 (54.5%) | 278 (58.4%) |  |
| **Gender** |  |  |  | 0.86 |
| **Female** | 28 (8.5%) | 13 (9.0%) | 41 (8.6%) |  |
| **Male** | 303 (91.5%) | 132 (91.0%) | 435 (91.4%) |  |
| **Race/Ethnicity** |  |  |  | 0.795 |
| **Black** | 14 (5.2%) | 7 (5.9%) | 21 (5.4%) |  |
| **Hispanic/Latino** | 12 (4.4%) | 5 (4.2%) | 17 (4.4%) |  |
| **Other** | 16 (5.9%) | 4 (3.4%) | 20 (5.2%) |  |
| **White** | 228 (84.4%) | 102 (86.4%) | 330 (85.1%) |  |
| **Missing** | 61 | 27 | 88 |  |
| **Bystander CPR?** |  |  |  | 0.001 |
| **No** | 101 (30.5%) | 24 (16.6%) | 125 (26.3%) |  |
| **Yes** | 230 (69.5%) | 121 (83.4%) | 351 (73.7%) |  |
| **Public AED Use?** |  |  |  | < 0.001 |
| **No** | 269 (81.3%) | 90 (62.1%) | 359 (75.4%) |  |
| **Yes** | 62 (18.7%) | 55 (37.9%) | 117 (24.6%) |  |
| **First Rhythm Type** |  |  |  | < 0.001 |
| **Non-shockable** | 164 (49.5%) | 18 (12.4%) | 182 (38.2%) |  |
| **Shockable** | 167 (50.5%) | 127 (87.6%) | 294 (61.8%) |  |
| **Survived To Hospital Admission?** |  |  |  | < 0.001 |
| **No** | 254 (76.7%) | 0 (0.0%) | 254 (53.4%) |  |
| **Yes** | 77 (23.3%) | 145 (100.0%) | 222 (46.6%) |  |
| **CPC_Score** |  |  |  |  |
| **CPC 1 or 2** | 0 | 141 (97.2%) | 141 (97.2%) |  |
| **CPC 3 or 4** | 0 | 4 (2.8%) | 4 (2.8%) |  |
| **Missing** | 331 | 0 | 331 |  |
| **Public/Private Golf Course** |  |  |  | 0.006 |
| **Private** | 116 (35.0%) | 71 (49.0%) | 187 (39.3%) |  |
| **Public** | 215 (65.0%) | 74 (51.0%) | 289 (60.7%) |  |
| **Witness** |  |  |  | < 0.001 |
| **Unwitnessed** | 88 (26.6%) | 12 (8.3%) | 100 (21.0%) |  |
| **Witnessed** | 243 (73.4%) | 133 (91.7%) | 376 (79.0%) |  |
| **EMS Response Interval** |  |  |  | 0.002 |
| **Mean (SD)** | 14.8 (37.0) | 10.7 (5.6) | 13.5 (30.8) |  |
| **Median (Q1, Q3)** | 11.2 (8.6, 14.8) | 10.0 (7.0, 12.4) | 10.8 (8.1, 14.1) |  |
| **Missing** | 67 | 23 | 90 |  |
